# Solution-Processed Efficient Blue Phosphorescent Organic Light-Emitting Diodes (PHOLEDs) Enabled by Hole-Transport Material Incorporated Single Emission Layer

**DOI:** 10.3390/ma14030554

**Published:** 2021-01-24

**Authors:** Taeshik Earmme

**Affiliations:** Department of Chemical Engineering, Hongik University, 94 Wausan-ro, Mapo-gu, Seoul 04066, Korea; earmme@hongik.ac.kr

**Keywords:** organic light-emitting diodes (OLEDs), solution-processing, hole-transport materials, fluorescence resonance energy transfer (FRET)

## Abstract

Solution-processed blue phosphorescent organic light-emitting diodes (PHOLEDs) based on a single emission layer with small-molecule hole-transport materials (HTMs) are demonstrated. Various HTMs have been readily incorporated by solution-processing to enhance hole-transport properties of the polymer-based emission layer. Poly(*N*-vinylcarbazole) (PVK)-based blue emission layer with iridium(III) bis(4,6-(di-fluorophenyl)pyridinato-*N*,C2′)picolinate (FIrpic) triplet emitter blended with solution-processed 1,1-bis[(di-4-tolylamino)phenyl]cyclohexane (TAPC) gave luminous efficiency of 21.1 cd/A at a brightness of 6220 cd/m^2^ with an external quantum efficiency (EQE) of 10.6%. Blue PHOLEDs with solution-incorporated HTMs turned out to be 50% more efficient compared to the reference device without HTMs. The high hole mobility, high triplet energy of HTM, and favorable energy transfer between HTM blended PVK host and FIrpic blue dopant were found to be important factors for achieving high device performance. The results are instructive to design and/or select proper hole-transport materials in solution-processed single emission layer.

## 1. Introduction

Considerable work has been carried out to research and develop high-performance phosphorescent materials and devices [1,2,3,4,5]. Highly efficient organic light-emitting diodes (OLEDs) enabled by phosphorescent emitters which can utilize both singlet and triplet excitons into photons could achieve 100% internal quantum efficiency. Most research for high-performance phosphorescent organic light-emitting diodes (PHOLEDs) has been focused on using small molecules as a host, performing co-evaporation to obtain a phosphorescent emissive layer [6,7,8,9,10]. Especially blue emitting PHOLEDs are more challenging than green or red PHOLEDs because the high triplet energy values of the excitons tend to be flown out from the emissive layer without any recombination process. A large progress has been made in the device efficiency of blue PHOLEDs by developing highly efficient charge-transport materials, high triplet energy host, and blue triplet emitters. A high external quantum efficiency of over 20% and a high current efficiency of over 30 cd/A have been already achieved by phosphorescent emission layer consist of a charge transport host doped with triplet emitters with multilayered device structures [11,12].

Although various studies have been conducted in order to realize highly efficient PHOLEDs, the emissive layers of these devices were typically made by delicate thermal evaporation to introduce triplet iridium(Ir)-complex emitters into hole transport hosts that require high-vacuum equipment and precise control of the co-evaporation rate, which leads to expensive fabrication cost [12,13]. Moreover, co-evaporation of the emissive layer using high vacuum chamber is not suitable for the large area device applications. Solution-processing fabrication methods provide attractive alternatives to those processed by vacuum deposition, mainly due to simplicity and economic features of solution-based manufacturing in ambient conditions [14,15,16]. Poly(*N*-vinylcarbazole) (PVK) is one of the well-known and most frequently used host materials for solution-processed phosphorescent emission layer (EML) blended with triplet emitter [17]. PVK has good processability with an amorphous structure which can prevent undesired nonradiative processes in the layer. In addition, ease of processing with good mechanical flexible property enables suitable candidates for large-area displays and next-generation solid-state lighting. While showing good physical and processing advantages, PVK has low hole mobility (only in the range of 10^−6^–10^−8^ cm^2^/V·s [18]). The relatively low hole mobility can be compensated by incorporation of other hole-transport materials (HTMs) in the emission layer. A simple solution blending of HTMs into the polymeric emission layer is possible; however, additional material should be selected carefully to enhance solution-processed blue PHOLEDs performance.

In this paper, we report a significant enhancement of the PHOLEDs performance enabled by additional solution-based incorporation of HTMs to the emission layer. Blue PHOLEDs based on a PVK emission layer doped with a FIrpic triplet emitter gave a high luminous efficiency of 21.1 cd/A at a brightness (luminance) of 6220 cd/m^2^ with an external quantum efficiency (EQE) of 10.6%. The PHOLEDs with incorporated TAPC show almost 50% increase in device efficiency compared to the device without HTMs (14.3 cd/A, 4870 cd/m^2^). However, not all the devices with incorporated HTMs show enhancement of device performance. In some cases, rather, decreased device performance was observed by the incorporation of HTMs. The results demonstrate that an incorporation of high mobility materials into the emission layer does not always guarantee improved device performance; rather, other properties such as the level of triplet energy of HTMs and overlapped spectra matching between donor HTM photoluminescence (PL) and the accepting triplet emitter absorption spectrum to facilitate Förster (Fluorescence) resonance energy transfer (FRET) are also very important. Four different HTMs have been selected, solution-added to the phosphorescent emission layer and investigated in order to understand the effect of different HTMs on the overall device performance.

## 2. Materials and Methods

### 2.1. Materials

**Figure 1:**
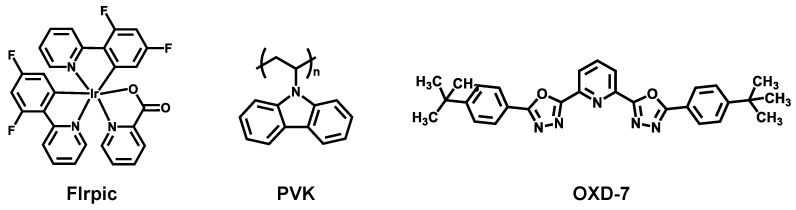
Chemical structures of materials used in phosphorescent emission layer; FIrpic blue triplet emitter, PVK host, and OXD-7.

**Figure 2:**
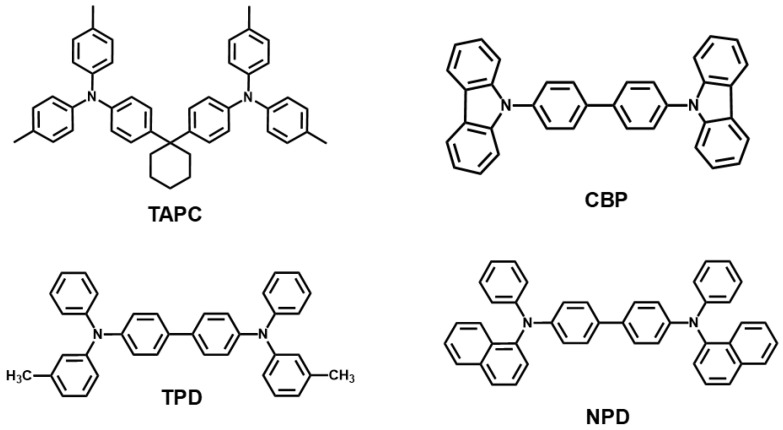
Chemical structures of various hole-transport materials incorporated in the EML.

**Figure 3:**
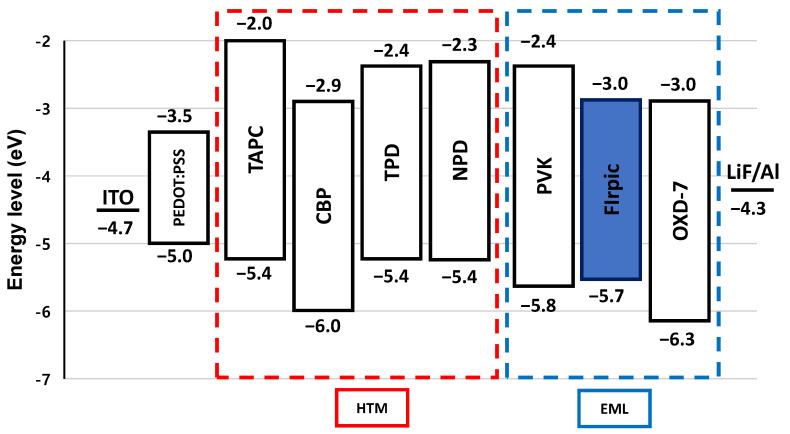
Energy levels of various HTMs and solution-processed blue EML components.

Pre-patterned glass-indium tin oxide (ITO) substrate with sheet resistance of 10 Ω/sq were purchased from AMG Ltd. (Uiwang, Korea). A solution of PEDOT:PSS (poly-(ethylenedioxythiophene)-polystyrenesulfonate, Clevios P VP Al 4083) was purchased from Hereus Co. (Hanau, Germany). The poly(*N*-vinyl carbazole) (PVK, M_w_ = 50,000–75,000, Sigma-Aldrich Korea, Merck, Seoul, Korea), 1,3-bis(2-(4-tert-butylphenyl)-1,3,4-oxadiazo-5-yl)benzene (OXD-7, Ossila Ltd., Sheffield, UK), and bis(3,5-difluoro-2-(2-pyridyl)phenyl-(2-carboxypyridyl)iridium (FIrpic, LumTec Co., Taipei City, Taiwan) used as received without further purification (Figure 1). Additional hole-transport materials; 1,1-Bis[(di-4-tolylamino)phenyl]cyclohexane (TAPC), 4,4′-Bis(*N*-carbazolyl)-1,1′-biphenyl (CBP), *N,N′*-Bis(3-methylphenyl)-*N,N*′-diphenylbenzidine (TPD) and *N,N′*--Bis(naphthalen-1-yl)- *N,N′*--bis(phenyl)benzidine (NPD) were all purchased from Ossila Ltd. All other chemicals were purchased from Sigma-Aldrich Korea. The chemical structures of all materials used here are shown in Figure 1 and Figure 2. Energy levels of materials used are specified in Figure 3.

### 2.2. Device Fabrication

The blue EML consisted of a blend of poly(*N*-vinyl carbazole) (PVK) 1,3-bis(2-(4-tert-butylphenyl)-1,3,4-oxadiazo-5-yl)benzene (OXD-7) with a ratio of PVK:OXD-7 = 60:40, wt/wt as a host and 10 wt.% FIrpic as the triplet emitting dopant. OXD-7 was added to enhance electron-transporting property of EML. A solution of PEDOT:PSS in water was filtered using 0.45 µm PVdF filter and spin-coated to deposit a ~30-nm hole-injection layer onto a pre-cleaned patterned ITO glass followed by thermal annealing at 150 °C for 30 min. Around 80-nm blue EML was generated by spin-coating of the PVK:OXD-7:FIrpic EML blends in chlorobenzene onto the PEDOT:PSS layer and vacuum dried at 100 °C. Devices with an additional HTMs (TAPC, CBP, TPD, or NPD) were dissolved into the EML blend solution before use. After drying, 1-nm of LiF and 100-nm Al was deposited sequentially.

### 2.3. Device Characterization

Each film thickness was separately measured by a film profilometer (Alpha-Step, KLA-Tencor, Milpitas, CA, USA). UV-Vis absorption spectra were collected on a model Lambda 365 UV/Vis/near-IR spectrometer (Perkin-Elmer, Waltham, MA, USA). The photoluminescence (PL) spectra were measured with a Perkin-Elmer FL 6500 fluorescence spectrophotometer. Electroluminescence (EL) spectra were analyzed using the same spectrophotometer described above. Current density-voltage (*J-V*) characteristics of the PHOLEDs were measured by using a 2450 Source Meter (Keithley, Beaverton, OR, USA). The brightness(luminance) was simultaneously obtained by using a chromameter (Model CS-1000A, Konica Minolta Holdings, Inc., Chiyoda, Tokyo, Japan). The device external quantum efficiencies (EQEs) were deducted from the current, brightness values and EL spectra assuming a Lambertian distribution same as previously reported procedures [16]. All the device fabrication and device measurement steps were performed under ambient condition.

## 3. Results and Discussion

**Figure 4:**
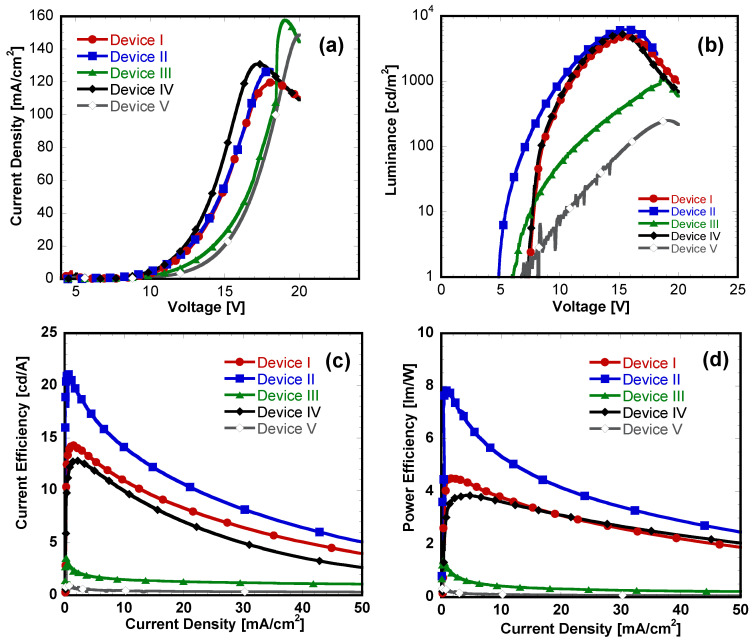
Device characteristics of the solution-processed blue PHOLEDs. (**a**) Current density-voltage (*J-V*); (**b**) luminance-voltage (*L-V*); (**c**) current efficiency-current density (*CE-J*); and (**d**) power efficiency-current density (*PE-J*) curves.

We fabricated blue PHOLEDs using a solution-deposited blue emission layer (EML) with four different hole-transport materials (HTMs) incorporated into the EML and without the HTMs as the reference. The EML containing poly(*N*-vinylcarbazole) (PVK) and 1,3-Bis[5-(4-tert-butylphenyl)-2-[1,3,4]oxadiazolyl]benzene (OXD-7) as the polymeric EML doped with the triplet blue emitter FIrpic as described in the Materials and Method Section (Section 2). Five sets of devices using different HTMs blended were fabricated to compare and investigate the effectiveness of additional HTMs to the EML in blue PHOLEDs: Device I (w/o HTM): ITO/PEDOT:PSS/PVK:OXD-7:FIrpic/LiF/Al; Device II (with TAPC): ITO/PEDOT:PSS/PVK:OXD-7:FIrpic:TAPC/LiF/Al; Device III (with TPD): ITO/PEDOT:PSS nm)/PVK:OXD-7:FIrpic:TPD/LiF/Al, Device IV (with CBP):ITO/PEDOT:PSS/PVK:OXD-7:FIrpic:CBP/LiF/Al; and Device V (with NPD):ITO/PEDOT:PSS/PVK:OXD-7:FIrpic:NPD/LiF/Al. Each device set contained four pixels on the same substrate with an area of 9 mm^2^ and the device parameters were averaged using the measured four values. The blend ratio of each HTM is initially fixed at 8 wt.% compared to the other components in the EML. The current density-voltage (*J-V*) and luminance (brightness)-voltage (*L-V*) characteristics of these sets of diodes are shown in Figure 4a,b.

Device I without additional HTM in the EML showed the maximum brightness of 4870 cd/m^2^ with a current efficiency (CE) value of 14.3 cd/A. The external quantum efficiency (EQE) was 7.2% while a turn-on voltage was 7.2 V. Towards our goal of achieving enhanced device performance, we incorporated additional HTM to the EML by solution-processing. Device II with TAPC in the EML showed the highest brightness of 6220 cd/m^2^ with much lower turn-on voltage of 4.8 V. The improved turn-on voltage of device II compared to the reference device I was presumably due to the facile injection of holes into the EML. The CE value of device II with TAPC remarkably increased to 21.1 cd/A with an EQE of 10.6%, which was almost 1.5 times higher than device I. This may be due to the high hole mobility of TAPC (~10^−3^ cm^2^/V·s) [19] and also lowering the energy barrier (HOMO level at −5.4 eV) for facilitating holes to be injected from adjacent PEDOT:PSS layer. Additionally, high LUMO value (−2.0 eV) of TAPC made possible to act as a good electron blocking material in the EML and thus led to enhancement of overall device performance. Device IV with CBP showed similar turn-on voltage (7.2 eV) and slightly higher maximum brightness of 5280 cd/m^2^ compared to the reference and increased device efficiency of 12.9 cd/A (EQE of 6.5 %). The higher performance resulted from the high hole mobility of CBP (~10^−3^–10^−4^ cm^2^/V·s) [20] similar as above TAPC added device. These blue PHOLEDs with additional TAPC or CBP demonstrates that the effectiveness of incorporating additional HTMs to the emission layer by solution-processing.

On the other hand, the overall device performance surprisingly decreased, which was unexpected in devices III and V. Although device III with TPD showed lower turn-on voltage (6.6 V) than device I, much lower maximum brightness of 1110 cd/m^2^ with a low CE value of 3.7 cd/A was observed. Even the drive voltage (at maximum brightness) of device III went up to 18.7 V, which is much higher value than the reference device (15.8 V). In spite of this, the HOMO level of −5.4 eV, which can facilitate hole-injection from PEDOT:PSS and relatively high hole mobility (~10^−4^ cm^2^/V·s) of TPD expected to increase overall hole-transporting property in the EML, the device performance significantly decreased. Furthermore, device V with NPD, which possesses very similar chemical and electronic structures as TPD [21], showed drastically decreased device performance with the maximum brightness only of 250 cd/m^2^ and device efficiency of 0.4 cd/A (EQE of 0.2%). These results imply that simply incorporating HTM with high hole mobility does not guarantee the enhancement of solution-processed PHOLEDs performance. All device characteristic parameters of the blue PHOLEDs are summarized in Table 1.

**Figure 5:**
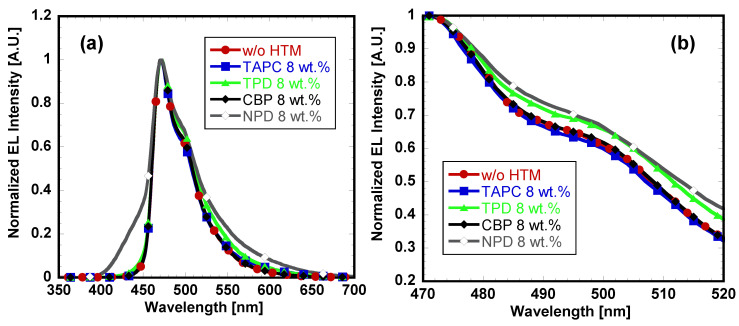
(**a**) EL spectra of the PHOLEDs; (**b**) EL spectra at 470 to 520 nm.

In order to investigate the resulted difference in device performances by using different HTMs, electroluminescence (EL) spectra of the PHOLEDs were measured at drive voltage (Figure 5a). The EL emission maximum peak of all the devices is identical at 472 nm which originated from FIrpic blue triplet emitter. The spectra associated with the devices using TAPC or CBP showed very similar line shape compared to the reference device without any incorporated HTMs. However, EL spectra of devices III and V with TPD and NPD, respectively, exhibited extra shoulder emission over 472 nm range (Figure 5b) which occurred presumably due to the different degree of microcavity effect [22]. The pronounced shoulder peak with TPD and NPD around 500 nm suggests that the position in exciton generation region in the EML is different from other three devices. We also note that the absolute EL intensities of devices III and V with TPD and NPD are significantly low compared to other device sets, which suggests the hindered transport of holes in the EML. This additional emission may disrupt the purity of blue color as well as decrease overall device performance. Moreover, EL spectrum of NPD showed broad emission peak in the range of 390 to 460 nm which could explain severely low performance of device V.

**Figure 6:**
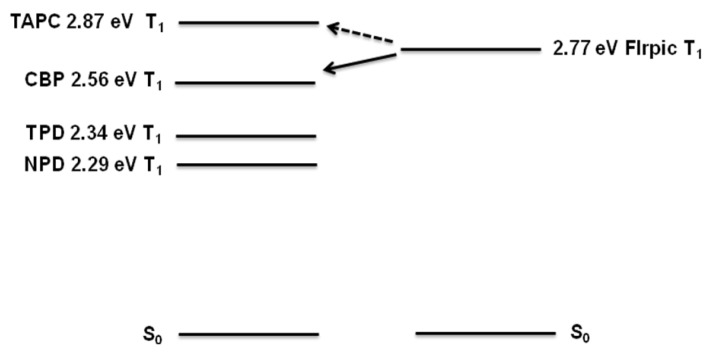
Comparison of the triplet energy values.

The different triplet energy values of incorporated HTMs in the EML could be another possible explanation of either increased or decreased PHOLEDs performance. Figure 6 shows the triplet energy values of incorporated HTMs and the blue triplet emitter FIrpic. Not only are low-lying HOMO level and high-lying LUMO levels of the host materials important to increase the exciton recombination in the EML confining holes and electrons, but so is a large triplet energy value of the materials in the layer. Typically, high triplet energy materials are used as adjacent layers next to EML to block triplet exciton from diffusing into other layers. Here, the triplet energy value of TAPC is 2.87 eV, which is sufficiently higher than FIrpic (2.77 eV), and could act as a good triplet exciton blocker [23] to increase device efficiency while CBP showed slightly lower value at 2.56 eV. This could explain the reason why although CBP has much low-lying HOMO level to confine holes than TAPC and similar hole mobility but exhibited slightly lower device performance such as lower maximum brightness and device efficiency compared to TAPC incorporated PHOLEDs (Table 1). In the case of TPD (2.34 eV) and NPD (2.29 eV), the triplet energy values are much lower than TAPC and CBP which are presumably not sufficient to well-confine FIrpic triplet excitons in the EML and thus a large portion of the triplet excitons be quenched [24]. The result demonstrates that the incorporation of high triplet energy material in the EML is essential to achieve high PHOLED performance.

**Figure 7:**
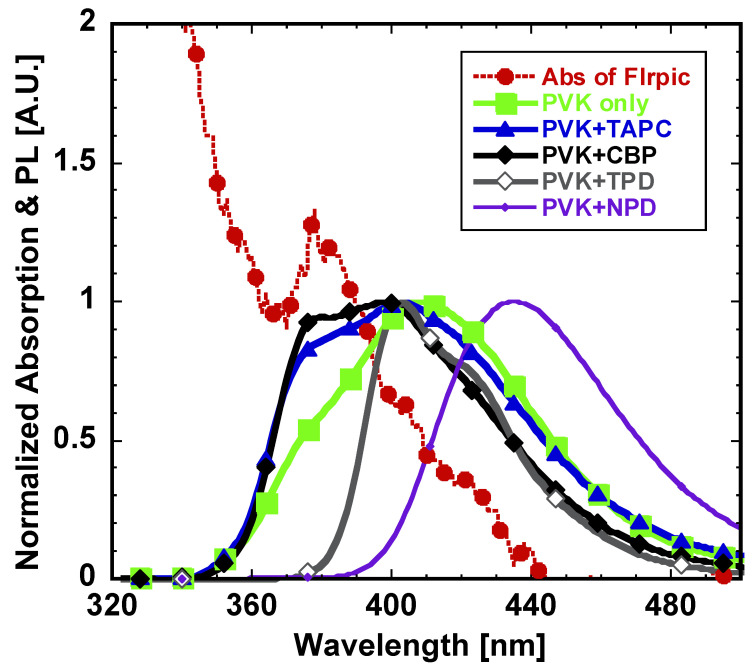
Normalized absorption spectrum of FIrpic blue triplet emitter and photoluminescent (PL) emission spectra of polymeric host layers.

Regarding the blue phosphorescent EML consisted of host-dopant system, an efficient energy transfer between the host and dopant should be considered. The EML used here comprised of a FIrpic dopant and PVK-based host with or without additional HTMs. From the blending system of EML used here, the long-range (~10 nm) Förster (Fluorescence) resonance energy transfer (FRET) is expected between polymeric host and the triplet dopant emitter. Figure 7 shows the photoluminescent (PL) emission spectrum of each polymeric host donor and the absorption spectrum of the dopant acceptor. FRET effectively occurs when the emission spectrum of the donor and absorption spectrum of the acceptor overlaps well, facilitating the energy transfer from excited to ground state of donor which may easily excite the acceptor group. The resonance efficiency of FRET mainly depends on the overlapped spectrum area; much larger overlapped area results in higher energy transfer rate [25].

As shown in Figure 7, The absorption spectrum of FIrpic acceptor has the most overlapped area with the emission spectra of PVK blended with TAPC, and similarly with CBP, while PVK’s only host emission shows rather smaller overlapped area with the absorption spectrum of the FIrpic dopant. Compared to the overlapped area between PVK only emission and FIrpic absorption spectra, that of PVK blended with TAPC and CBP showed 21.1% and 22.8% higher overlapped area, respectively. However, PVK blended with TPD emission spectrum showed much smaller overlapped area, 41.7% decreased value compared to PVK only emission, which correlates with the low device efficiency as shown above. Likewise, PVK blended NPD has the least overlapping spectrum area only of 29.8 % compared to the PVK only which well corresponds to the lowest device performance using the EML with NPD. The photophysical study described here suggests that the effective FRET between the polymeric host and the emitter dopant is important to achieve high performance of PHOLEDs.

## 4. Conclusions

In summary, highly efficient blue phosphorescent OLEDs were realized by the incorporation of hole-transport materials to the solution-processed single emission layer. Various hole-transport materials were demonstrated to enhance hole-transport property of a PVK-based emission layer. The blue PHOLEDs with TAPC or CBP hole-transport materials (HTMs) showed clear improvement in device performance compared to the reference device without additional hole-transport materials, while the devices with TPD or NPD resulted in significantly decreased efficiency. The blue PHOLEDs with TAPC exhibited almost a 150% increase in device efficiency (21.1 cd/A) and maximum brightness (6220 cd/m^2^), whereas the device performance with NPD largely dropped down to 0.4 cd/A and 250 cd/m^2^. Although all additional HTMs possess much higher hole mobilities (~10^−3^–10^−4^ cm^2^/V·s) compared to PVK host polymers (~10^−6^–10^−8^ cm^2^/V·s), the huge difference in device performance observed by incorporating hole-transport materials presumably originated from different electronic structures of incorporated materials. The EL spectra of the PHOLEDs showed that using TPD or NPD led to undesired EL emission, which affected overall device efficiency. This undesired emission could be caused by low triplet energy values of TPD and NPD relative to that of FIrpic blue triplet emitter which are not suitable to achieve good confinement of triplet excitons in the blue EML. In addition, efficient energy transfer between the solution-processed host and guest emitter should be considered to realize high device efficiency. These results demonstrate that the various small-molecule HTMs can readily be processed from solution to achieve high performance solution-processed blue PHOLEDs with a single emission layer. These results could be instructive in the design of HTMs in solution-processed PHOLEDs for next-generation light-emitting displays and solid-state lighting.

## Figures and Tables

**Table 1:** Device characteristics of the blue phosphorescent OLEDs.

Device	Incorporated HTM	Turn-On Voltage ^1^ (V)	Drive Voltage (V)	Current Density ^2^ (mA/cm^2^)	Max Luminance (cd/m^2^)	Device Efficiency (CE (cd/A)), (EQE (%))
Device I (w/o HTM)	None	7.2 ± 0.1	15.8 ± 0.1	75.9 ± 2.4	4870 ± 30	14.3 ± 0.07, 7.2 ± 0.04
Device II (w/ TAPC)	TAPC	4.8 ± 0.1	15.8 ± 0.1	73.0 ± 2.3	6220 ± 30	21.1 ± 0.11, 10.6 ± 0.05
Device III (w/TPD)	TPD	6.6 ± 0.1	18.7 ± 0.1	150.4 ± 2.7	1110 ± 10	3.7 ± 0.03, 1.9 ± 0.02
Device IV (w/CBP)	CBP	7.2 ± 0.1	15.4 ± 0.1	81.3 ± 2.5	5280 ± 20	12.9 ± 0.06, 6.5 ± 0.04
Device V (w/NPD)	NPD	6.9 ± 0.1	19.1 ± 0.1	128.0 ± 3.3	250 ± 10	0.4 ± 0.02, 0.2 ± 0.01

^1^ Turn-on voltage at 1 cd/m^2^; ^2^ Current density at maximum luminance.

## Data Availability

Data is contained within the article.
